# The protective effect of kirenol in osteoarthritis: an in vitro and in vivo study

**DOI:** 10.1186/s13018-022-03063-y

**Published:** 2022-04-01

**Authors:** Wei Hu, Chao Mao, Weibin Sheng

**Affiliations:** Department of Spine Surgery, Xinjiang Medical University Affiliated First Hospital, Urumqi, Xinjiang China

**Keywords:** Kirenol, Osteoarthritis, Chondrocytes, Inflammation, NF-κB

## Abstract

**Background:**

Osteoarthritis (OA) is a chronic degenerative disease, its main characteristic involves articular cartilage destruction and inflammation response, absent of effective medical treatment. Our current research aimed to explore anti-inflammatory effect of kirenol, a diterpenoid natural product compound, in the development of OA and its potential molecular mechanism through in vitro and in vivo study.

**Methods:**

In vitro, chondrocytes were pretreated with kirenol for 2 h before IL-1β stimulation. Production of NO, PGE2, TNF-α, IL-6, aggrecan, collagen-II, MMP13and ADAMTS5 were evaluated by the Griess reaction and ELISAs. The mRNA (aggrecan and collagen-II) and protein expression (COX-2, iNOS, P65, IκB, PI3K, AKT) were measured by qRT-PCR and Western blot respectively. Immunofluorescence was used to assess the expression of collagen-II and P65. The in vivo effect of kirenol was evaluated in mice OA models induced by destabilization of the medial meniscus (DMM).

**Results:**

We found that kirenol inhibited IL-1β-induced expression of NO, PGE2, TNF-α, IL-6, COX-2, iNOS, ADAMTS-5. Besides, kirenol remarkably decreased IL-1β-induced degradation of aggrecan and collagen-II. Furthermore, kirenol significantly inhibited IL-1β-induced phosphorylation of PI3K/Akt and NF-κB signaling. In vivo, the cartilage in kirenol-treated mice exhibited less cartilage degradation and lower OARSI scores.

**Conclusions:**

Taken together, the results of this study provide potent evidence that kirenol could be utilized as a potentially therapeutic agent in prevention and treatment of OA.

## Introduction

Osteoarthritis (OA) is a prevalent degenerative disease, which could lead to severe joint pain and disability especially in older people [1]. Pathological findings in OA include articular cartilage destruction, subchondral bone remodeling and synovial inflammation [2]. Previous studies indicated that a number of factors, such as genetics, aging, gender, obesity, and joint trauma, may contribute to the occurrence and development of OA [3, 4]. Although the specific mechanism of OA remains unknown, several studies have shown that the pro-inflammatory cytokines are key factors involved in the development of OA, especially Interleukin-1β (IL-1β) [5, 6]. And increasing level of IL-1β was found in synovial membrane and synovial fluid of OA patients [7, 8]. Furthermore, Kobayashi and Abramson demonstrated that accumulated IL-1β exerts its damaging effects via significantly increasing the generation of inflammatory mediators and catabolic factors, including nitric oxide (NO), prostaglandin E2 (PGE2), thrombospondin motifs (ADAMTS) and matrix metalloproteinases (MMPs), which caused the chondrocytic dysfunction and destroyed the extracellular matrix (ECM) composition [9, 10]. Therefore, these finding indicate that inhibiting the IL-1β-induced inflammatory responses may be a promising strategy to delay the OA development.

Results from different studies have noted that the nuclear factor kappa B (NF‐κB) pathways is important to pathogenesis and progression of OA and strongly associated with inflammatory response and catabolism induced by IL‐1β [11, 12]. During normal situation, NF-κB is located in the cytoplasm as an inactive state, connected with IκBα, an inhibitory subunit. After IL-1β stimulation, IKK (IκB kinase) is activated by membrane proximal events, causing the IκBs phosphorylation and following degraded in the cytoplasm, further leading to NF‐κB p65 phosphorylation and translocation from the cytoplasm to the nucleus, binding with corresponding sites during promoter regions, which modulates the translation of related downstream devastative gene, such as iNOS, COX-2, MMPs and ADAMTs [13, 14]. In addition, prior studies have showed that PI3K/AKT pathway might be one of the main upstream targets of activation of NF-κB pathway, the underlying mechanism may depend on the phosphorylation of IκBα and p65 [15]. Thus, strategies aiming at the inhibition of PI3K/AKT/NF-κB pathway hold promise in attenuating the OA progression.

Kirenol is the major active diterpenoid component extracted from the Chinese herbal medicine Siegesbeckiae, which has potent anti-inflammatory properties [16]. A recent research has proved the wound healing property of kirenol through the suppression of inflammatory response and matrix metalloproteinase expressions [17]. Furthermore, anti-inflammatory effect of kirenol has been verified in diabetic cardiomyopathy by means of the regulation of NF-κB signaling [18]. Qian et al. has demonstrated that kirenol could inhibit NF-κB activity by upregulating nuclear annexin-1 which interacted with NF-κB, and then reduced cytokines expression and thereby attenuated synovial inflammation of collagen-induced arthritis [19]. Additionally, Wang et al. has proved that kirenol may exhibit therapeutic modality for thyroid cancer through PI3K/AKT and MAP kinase signaling pathways [20]. However, relevant study of the effect of kirenol as an inhibitor of inflammatory response in the OA development remains limited. Thus, current research was to explore the anti-inflammatory role and potential molecular mechanism of kirenol on chondrocytes in vitro and mouse OA model in vivo. This might set a precedent for the application of kirenol-derived drugs in the potential treatment of OA.

## Materials and methods

### Reagents

Kirenol (purity > 98%) was obtained from Solarbio (Beijing, China), and Recombinant human IL-1β was purchased from PeproTech (NJ, USA). Collagenase type II and dimethylsulfoxide (DMSO) were purchased from Sigma Chemical Co. (MO, USA). Primary antibodies against collagen II, COX-2 and iNOS were obtained from Abcam (Cambridge, UK). Primary antibodies against p65, IkBα, AKT and p-AKT were obtained from Cell Signaling Technology (MA, USA). β-actin and Lamin B antibodies were obtained from Sigma Aldrich (St Louis, MO, USA). Alexa Fluor®488 labeled and Goat Anti-Rabbit IgG second antibody were purchased from Jackson ImmunoResearch (West Grove, PA). The 4', 6-diamidino-2-phenylindole (DAPI) was purchased from Beyotime (Shanghai, China). The cell culture reagents were obtained from Gibco (NY, USA).

### Primary mice chondrocytes isolation and culture

Ten 2-week-old C57BL/6 mice (5 males and 5 females) were euthanized through overdose of sodium pentobarbital as previously described [21]. Briefly, the cartilage was isolated from the knee joint of mice by a dissecting microscope under aseptic condition, then cartilage was cut into pieces and dissolved in 2 mg/ml (0.1%) type II collagenase for 4–6 h at 37 °C. Next, digests were centrifuged at 1000 rpm for 5 min, supernatant fluid was removed and resuspended in DMEM/F12 with 10% fetal bovine serum (FBS) as well as 1% penicillin/streptomycin antibiotics, further seeding into the 6-well plate at the appropriate environment of 5% CO_2_ at 37 °C. The 0.25% Trypsin–EDTA solution was added in chondrocytes when cell density growing to 80–90% confluence. The second-passage chondrocytes were selected for the following test to avoid phenotype loss.

### Mice OA model

Fifty 8-week-old C57BL/6 male wild-type (WT) mice were acquired from Animal Center of Chinese Academy of Sciences, Shanghai, China. The protocol for animal care and use complied with The Guide for the Care and Use of Laboratory Animals of the National Institutes of Health and was ratified by the Animal Care and Use Committee of Xinjiang Medical University. OA model was built through surgically destabilizing the medial meniscus (DMM) described in previous study [22]. Briefly, mice were anesthetized with 2% (w/v) pentobarbital (40 mg/kg) intraperitoneally, and then joint capsule was carved medial to the patellar tendon of the right knee, medial meniscotibial ligament was cut using a microsurgical scissor. After recovering from the surgery, mice were randomized into three groups: sham group, vehicle group, as well as kirenol-treated group.

### Experimental design

In vitro study, chondrocytes were stimulated with IL-1β (10 ng/mL), alone or combined with pretreatment of kirenol at different concentration (10, 20 and 40 µM). In the control group, chondrocytes were treated nothing except daily medium change. chondrocytes were collected after 24 h incubation.

Surgical DMM model was applied to evaluate the therapeutic effect of kirenol in vivo, mice in kirenol-treated group received kirenol (50 mg/kg/day) and administrated once a day by intraperitoneal injection for consecutive eight weeks. Meanwhile, mice in sham group were treated with same dose of physiological saline. All mice were sacrificed at eight weeks post-surgery, and knee joint specimens were harvested for further historical assessment.

### Cell viability

The cytotoxic effect of kirenol on chondrocytes was determined by CCK-8 kit in accordance with manufacturer’s instruction. The second-passage chondroyctes were incubated into 96-well plates (5 × 10^3^ cells per well) for 24 h, and pretreated with different concentrations (0, 5, 10, 20, 40 and 80 μM) of kirenol for 24 h and 48 h. Then the cells were washed with PBS and each well was added with 10 μl CCK-8 solution and cultivated at 37 °C for 2 h. A microplate reader (Leica Microsystems, Germany) was adopted to detect the absorbance of each well at 450 nm wavelength. All experiments were performed six times.

### Griess reaction and ELISA test

Griess reagent was utilized to measure the production of NO. The IL-6, TNF-α, PGE2, aggrecan, collagen II, MMP13, and ADAMTS-5 concentrations in cell suspensions were evaluated using the common ELISA kits in accordance with the company’s guidelines. All examines were performed six times.

### Western blot analysis

Radioimmunoprecipitation assay (RIPA) buffer with 1 mM phenylmethylsulfonyl fluoride (PMSF) was applied to isolate the whole cell proteins from chondrocytes. The nuclear and cytoplasmic proteins were isolated respectively by mean of the Nuclear and Cytoplasmic Protein Extraction Kit. Then lysates on ice were treated with ultra-wave sonication followed by centrifugation at 12,000 rpm at 4 °C for 30 min. Furthermore, protein concentration was measured via the BCA protein assay. About 20 ng protein from each sample was separated by sodium dodecylsulfate-polyacrylamide gel electrophoresis (SDS PAGE), followed by transferring to a polyvinylidene fluoride membrane (Bio-Rad, USA). Later, membranes were blocked for 2 h with 5% nonfat milk, and probed with the primary antibodies specific for β-actin (1:5000), Lamin B (1:5000), COX-2 (1:1000), iNOS (1:1000), IkBα (1:1000), p65 (1:1000), AKT (1:1000) and p-AKT (1:1000) at 4 °C overnight. After washing three times with TBST, the membranes were incubated with corresponding secondary antibodies (1:5000) for 2 h at room temperature. Eventually, blots were visualized by enhanced chemiluminescence (ECL) kit while blots intensity was measured by an observer blinded to the experimental group through Image Lab 3.0 software (Bio-Rad). β-Actin and Lamin B were performed as the standard proteins.

### Real-time polymerase chain reaction (RT-PCR)

Total RNA was isolated using TRIzol (Invitrogen) according to the manufacturer’s instructions. cDNA was synthesized from 1 μg of RNA via the One Step RT-PCR Kit (TaKaRa). Quantitative real-time PCR were performed using the iQTM SYBR Green Supermix PCR kit with the iCycler apparatus system (Bio-Rad). The primer sequences were as follows: for collagen II and aggrecan, GAPDH was used as the invariant housekeeping gene internal control. The primers of collagen II and aggrecan were designed using NCBI Primer-Blast Tool, and were as follows: collagen II (F) 5′-CTCAAGTCGCTGAACAACCA-3′, (R) 5′-GTCTCCGCTCTTCCACTCTG-3′; aggrecan (F) 5′-AAGTGCTATGCTGGCTGGTT-3′, (R) 5′-GGTCTGGTTGGGGTAGAGGT-3′; GAPDH (F) 5′-TCTCCTCTGACTTCAACAGCGAC-3′, (R) 5′-CCCTGTTGCTGTAGCCAAATTC-3′.

### Immunofluorescence

For immunofluorescence staining, chondrocytes (4 × 10^5^ cells/ml) were added into six-well plates, cultured in serum-starved medium overnight. Afterwards, chondrocytes were stimulated with 10 ng/ml IL-1β alone or combined with 40 μM kirenol for 24 h. After which, cells were washed three times using PBS and fixed with 4% paraformaldehyde for 15 min, and washed with PBS again. Later, 0.1% Triton X-100 was applied for cells and nuclear membranes permeabilization for 15 min at room temperature. Then cells were treated with 10% goat serum for blocking for 1 h at room temperature and followed by PBS washing, and probed with the primary antibodies specific for collagen II (1:200) and p65 (1:200) overnight at 4 °C. After washing three times with TBST, cells were incubated with Alexa Fluor® 488-labelled conjugated secondary antibodies (1:1000) for 1 h at indoor temperature, and then labeled with DAPI for nuclear staining for 5 min. Finally, six visual fields were randomly selected for microscopic observation using a fluorescence microscope (Olympus Inc, Japan), and fluorescence intensity was measured by an observer blinded to the experimental group with through Image J software 2.1 (Bethesda).

### Histological assessment

The mice were sacrificed at 8 weeks post-surgery and knee joint specimens were collected. For histological assessment, the knee joint specimens were fixed in 4% paraformaldehyde for 24 h, and then decalcified in 10% ethylenediaminetetraacetic acid (EDTA) solution for 1 month. Samples then were dehydrated and embedded in paraffin, sectioned into 5 μm sagittal sections for further experiments. Each selected section was stained with Safranin O-fast green (SO). The stained sections were visualized using a microscope. Cartilage and subchondral bone cellularity and morphology were then evaluated using the Osteoarthritis Research Society International (OARSI) scoring, which was described previous to determine the severity of joint cartilage damage [23].

### Immunohistochemical analysis

The collagen II, MMP13, AKT and p-AKT level in vivo were assessed through immunohistochemical staining. Paraffin-embedded sections were deparaffinized by xylene and rehydrated by the gradient of ethanol, sections then were treated with 3% hydrogen peroxide for endogenous peroxidase blocking. Thereafter, 0.4% pepsin in 5 mM HCl was added to the section for antigen retrieval for 20 min at 37 °C. Later, sections were blocked using 5% bovine serum albumin for 30 min at room temperature and then probed with primary antibodies specific for collagen II (1:100), MMP13 (1:100), AKT (1:1000) and p-AKT (1:1000) at 4 °C overnight. After washing, the sections were treated with HRP-conjugated secondary antibodies at room temperature for 2 h and followed by hematoxylin incubation for nucleus staining. Finally, images were detected by microscope while the quantitative assessment was accomplished by an observer blinded to the experimental group through Image-Pro Plus software (Version 6.0). Six sections from each group were selected for quantitative evaluation.

### Statistical analysis

The experiments were repeated at least six times. Data were presented as the means ± standard deviation (SD). Statistical analyses were conducted through SPSS software version 20.0 (Chicago, USA). One-way analysis of variance (ANOVA) was used for analysis of two or more groups while Tukey’s test was adopted for comparison between groups. Nonparametric data were compared via the Kruskal–Wallis H test. *P* < 0.05 was considered statistically significant.

## Results

### Effect of kirenol on chondrocytes cytotoxicity

**Fig. 1:**
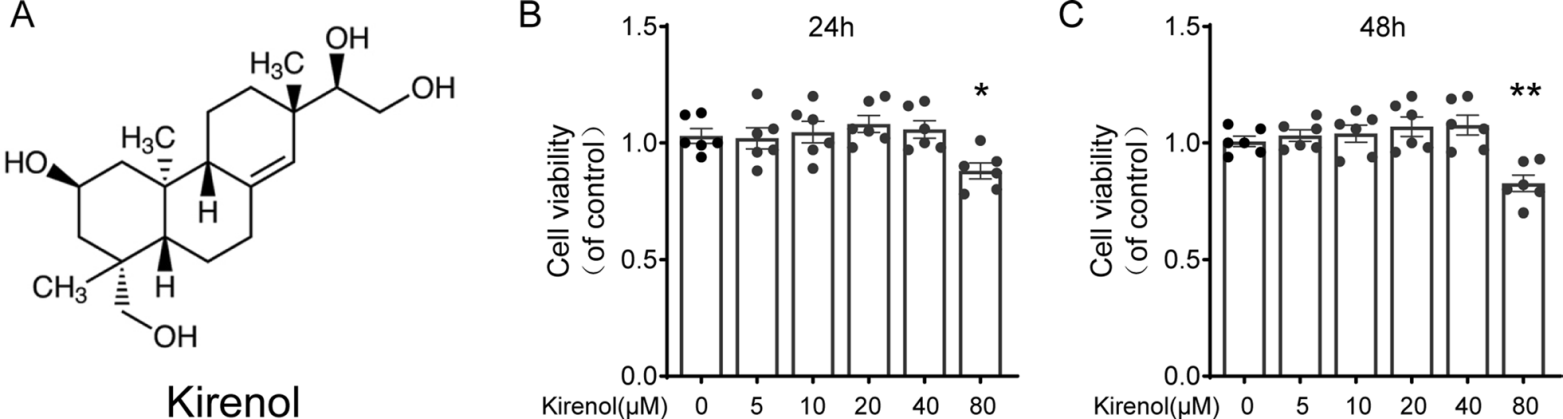
Effect of kirenol on the cell viability of chondrocytes (**A**). Chemical structure of kirenol. **B**, **C** The cytotoxic effects of kirenol (0, 5, 10, 20, 40 and 80 μM) on chondrocytes were determined at various concentrations for 24 and 48 h using a CCK8 assay. The data presented are the means ± S.D. *P < 0.05, **P < 0.01 vs. control group, *n* = 6

The chemical structure of kirenol was shown in Fig. 1A. When chondrocytes were treated with kirenol at the ascending concentrations (0, 5, 10, 20, 40 and 80 μM) for 24-48 h and CCK-8 assay was used to assess the cytotoxic effects of kirenol on chondrocytes. Results showed that optimal viability was achieved when chondrocytes were treated using 40 μM kirenol in 24 h or 48 h (Fig. 1B, C). Thus, non-cytotoxic concentrations of kirenol at 10, 20 or 40 μM were selected for the following experiments.

### Effect of kirenol on the level of IL-1β-induced inflammatory mediators in chondrocytes

**Fig. 2:**
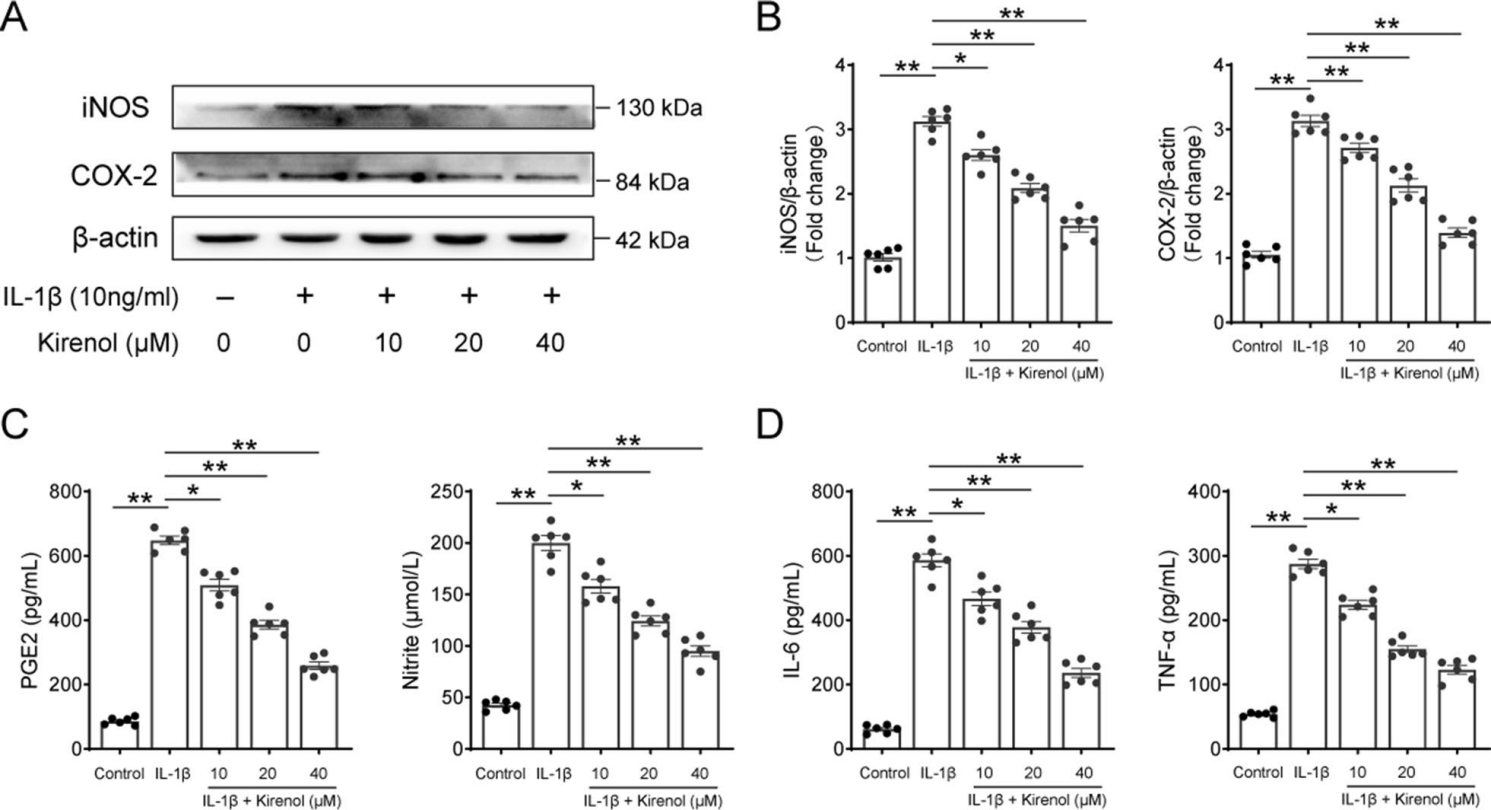
Effect of kirenol on IL-1β–stimulated inflammatory cytokines in chondrocytes. **A**, **B** The protein expression and quantification analysis of iNOS and COX-2 on chondrocytes treated as above were measured by western blot. **C**, **D** The protein level of PGE2, NO, TNF-α and IL-6 production on chondrocytes treated as above were assessed by ELISA. The data presented are the means ± S.D. *p < 0.05, and **p < 0.01, one-way AVOVA, *n* = 6

We next explored the anti-inflammatory effect of kirenol in chondrocytes, the expression of related inflammatory cytokines was detected using Western blot and ELISA. Through Western blot and its quantification analysis, we found that kirenol could inhibit the protein level of COX-2 and iNOS, which were up-regulated by IL-1β stimulation (Fig. 2A, B). Moreover, the expression of endogenous PGE2, NO, IL-6 and TNF-α in chondrocytes were increased after IL-1β treatment. From the results of Fig. 2C and D, kirenol was shown to significantly decrease PGE2, NO, IL-6 and TNF-α level in dose-dependent manner using ELISA kit. Thus, these findings suggest that kirenol could restrain the inflammatory response in chondrocytes within IL-1β stimulation.

### Effect of kirenol on IL-1β-induced ECM degradation in chondrocytes

**Fig. 3:**
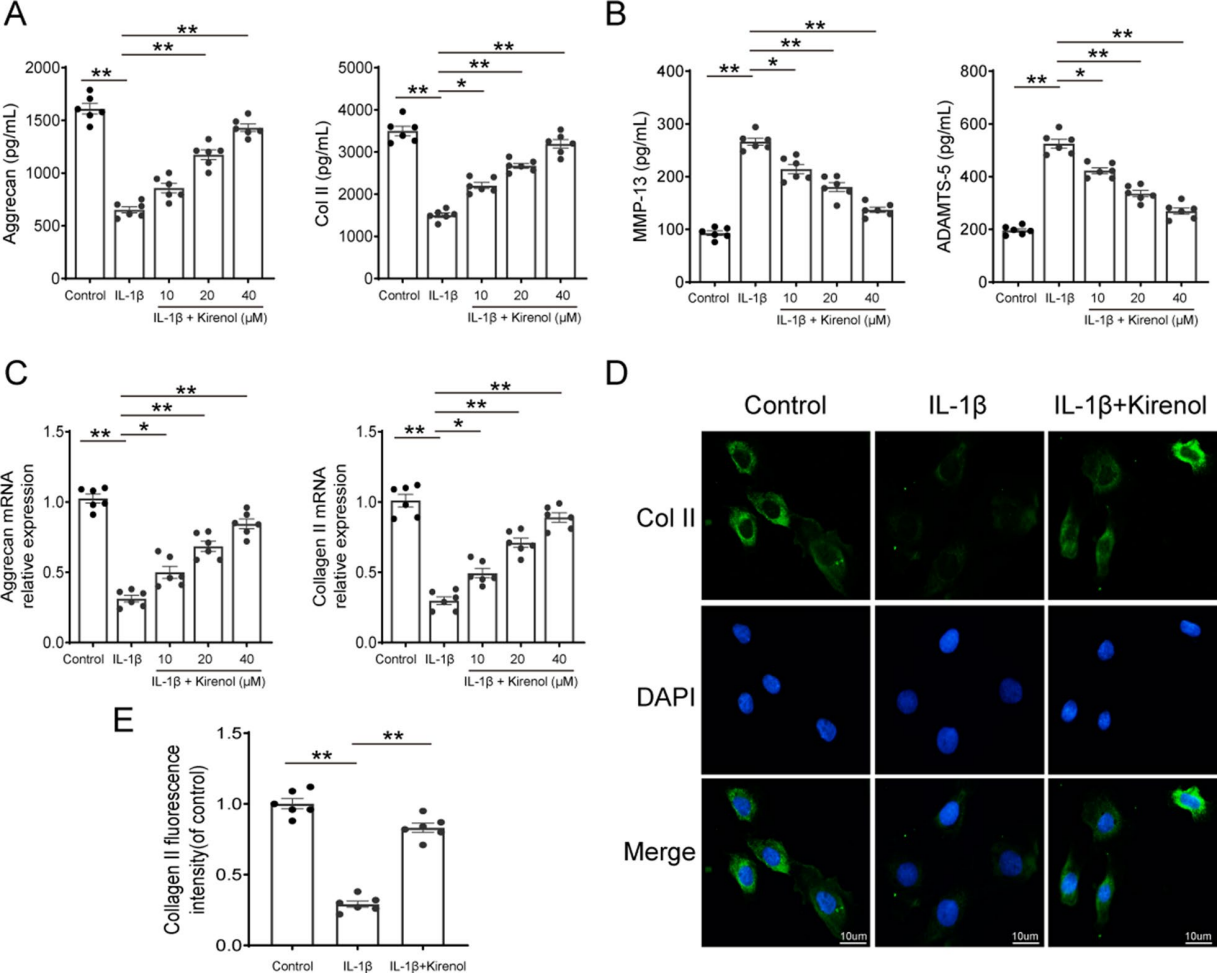
Effect of kirenol on IL-1β-induced extracellular matrix (ECM) degradation in chondrocytes. **A**, **B** The protein level of Col II, aggrecan, MMP-13 and ADAMTS5 production on chondrocytes treated as above were detected by ELISA. **C** RT-PCR results of mRNA expressions of aggrecan and collagen II. **D** The representative fluorescence image of collagen II with DAPI (nuclei); scale bar: 10 μm. **E** The fluorescence intensity of collagen II was quantified by Image J software. The data presented are the means ± S.D. *p < 0.05, and **p < 0.01, one-way AVOVA, *n* = 6

The balance of ECM synthesis and degradation has been considered as a key factor for chondrocytes function. We next explored the effect of kirenol on IL-1β-induced ECM degradation in chondrocytes by measuring the expression of aggrecan, collagen II, MMP13 and ADAMTS5. We found that IL-1β treatment caused the marked increase of ADAMTS5 and MMP13 expression and obvious reduction of aggrecan and collagen II production, and kirenol pre-treatment reversed these destructive effects in a dose-dependent manner (Fig. 3A, B). mRNA detection of aggrecan and collagen II further confirmed the above results (Fig. 3C). In addition, immunofluorescence result of collagen II shown in Fig. 3D and E supported that kirenol increased collagen level induced by IL-1β. Therefore, these results demonstrate that kirenol could slow down the degradation of ECM in IL-1β-stimulated chondrocytes.

### Effect of kirenol on IL-1β-induced NF-κB pathway activation in chondrocytes

**Fig. 4:**
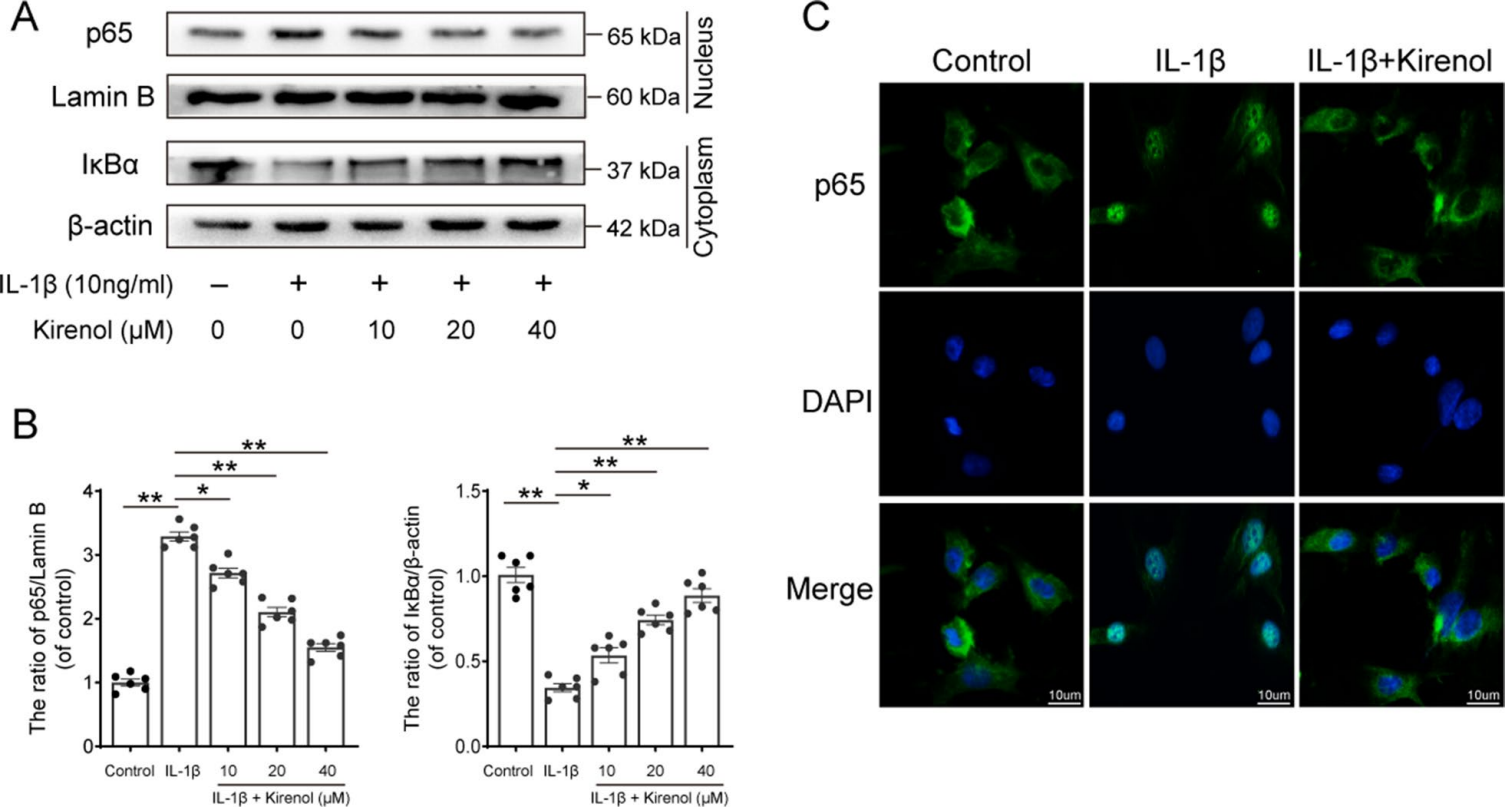
Effect of kirenol on IL-1β-induced NF-κB signaling pathway activation. The protein expression of p65 in nuclear and IκBα in cytoplasm on chondrocytes treated as above were visualized by western blot (**A**), and quantified in (**B**). **C** The nuclei translocation of p65 was detected by the immunofluorescence combined with DAPI staining for nuclei (scale bar: 10 µm). The data presented are the means ± S.D. *p < 0.05, and **p < 0.01, one-way AVOVA, *n* = 6

NF-κB pathway plays a vital role in the inflammatory response. Thus we detected the cytoplasmic IκBα degradation and p65 translocation in chondrocytes. The results showed that IL-1β stimulation significantly led to degradation of IkBa and up-regulated nuclear level of p65, while kirenol strongly inhibited these effects in a dosage dependent manner (Fig. 4A, B). Furthermore, immunofluorescence results revealed that IL-1β was sufficient to induce translocation of p65 from the cytoplasm to the nucleus, in accordance with the results of western blot. Not surprisingly, this effect was significantly prevented by kirenol pre-treatment (Fig. 4C). Altogether, these data demonstrated that kirenol pre-treatment could attenuate IL-1β-induced NF-κB pathway activation in chondrocytes.

### Effect of kirenol on IL-1β-induced PI3K/Akt pathway activation in chondrocytes

**Fig. 5:**
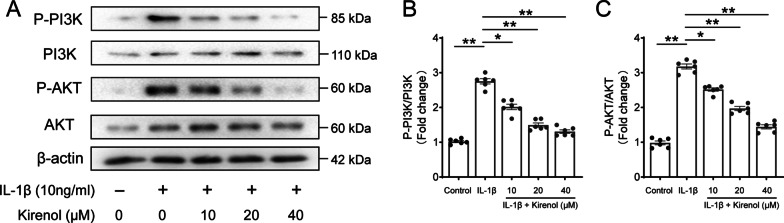
Effect of kirenol on IL-1β-induced PI3K/Akt signaling pathway activation. The phosphorylation level of PI3K and AKT on chondrocytes treated as above were determined by western blot (**A**) and quantification analysis (**B**, **C**). The data presented are the means ± S.D. *p < 0.05, and **p < 0.01, one-way AVOVA, *n* = 6

PI3K has been reported to involve in the NF-κB pathway activation and IL-1β-mediated inflammatory response. Western blot analysis was conducted to examine the effect of kirenol on IL-1β-stimulated phosphorylation of PI3K and Akt, and subsequently verify the anti-inflammatory molecular mechanisms of kirenol. The results found that the phosphorylation level of PI3K and Akt were obviously up-regulated induced by IL-1β treatment in comparison to the control group, as presented in the Fig. 5A, B and C, kirenol strongly suppressed IL-1β-stimulated PI3K/Akt signailing activation. Altogether, these data demonstrated that kirenol may exert its anti-inflammatory effect through PI3K/Akt signaling inhibition in IL-1β-mediated chondrocyte.

### Kirenol alleviates development of OA in the DMM mouse model

**Fig. 6:**
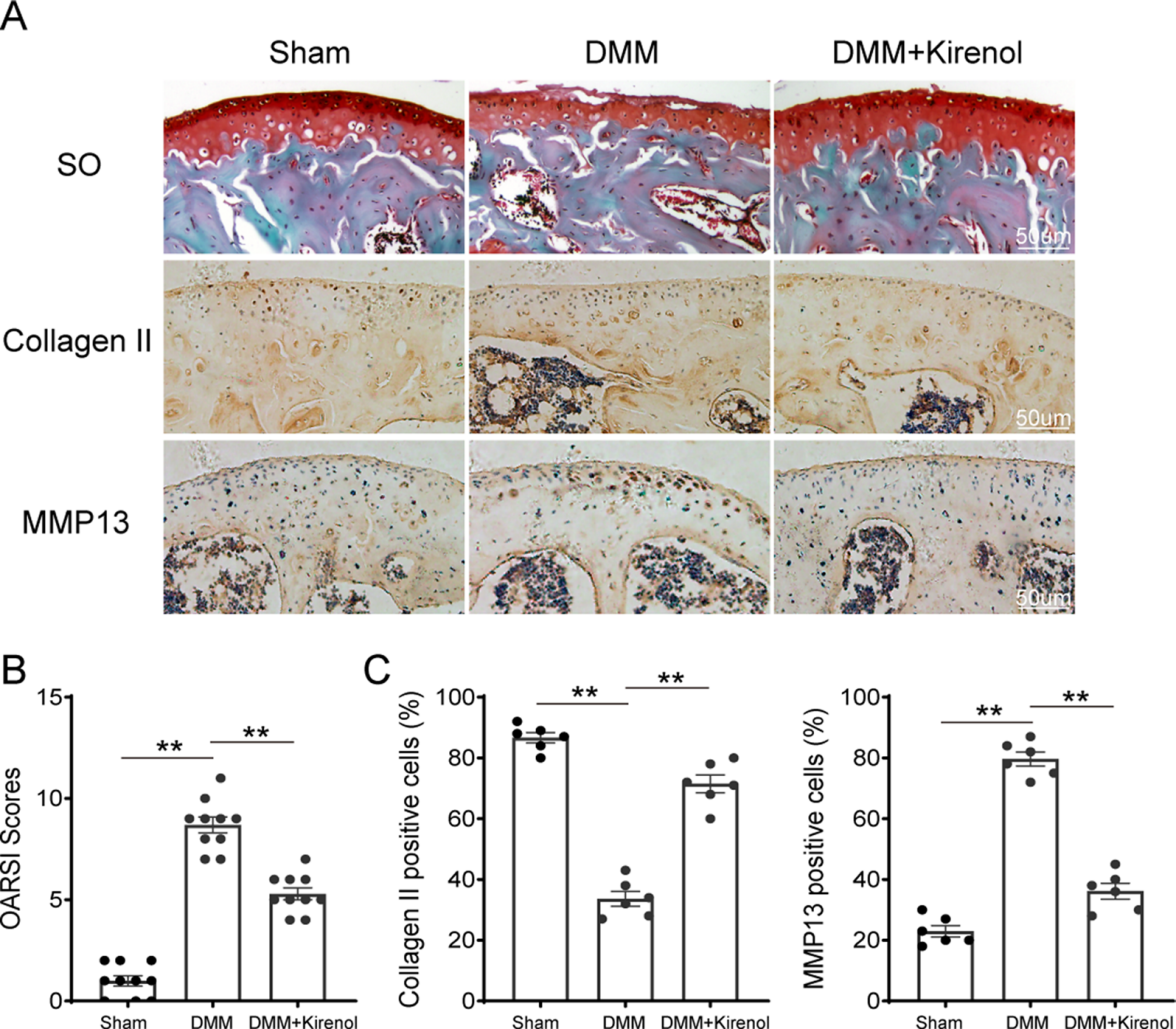
Effect of kirenol on OA development in mouse DMM model in vivo. **A** Typical S–O staining of cartilage and subchondral cortical bone from the different experimental groups at 8 weeks post-surgery (scale bar: 50 µm), and immunohistochemical staining of collagen II, MMP13 expression in the cartilage samples (scale bar: 50 µm). **B** Diagram showing the OARSI scores of the cartilage. **C** The percentages of collagen II and MMP13 positive cells in each section were quantified by Image Pro Plus. The data presented are the means ± S.D. *p < 0.05, and **p < 0.01, one-way AVOVA, *n* = 10 for cartilage OARSI scores, *n* = 6 for immunohistochemical staining

We also explored the protective effect of kirenol in surgically induced mouse model of DMM in vivo. SO staining was applied for histological and morphological analysis of cartilage, and the results were showed in Fig. 6A. OA group showed apparent cartilage abrasion, noticeable cartilage hypocellularity and vast proteoglycan loss compared to the sham group, in contrast, smoother cartilage surface and lessened proteoglycan loss were observed after kirenol treatment. In addition, Osteoarthritis Research Society International (OARSI) scores were also applied for the quantitative assessment. The OARSI scores in OA group were higher than that in the sham group, while kirenol group showed lower OARSI scores compared to the OA group, which was consistent with the results of SO staining (Fig. 6B).

Furthermore, immunohistochemical staining was carried out to measure the level of collagen II and MMP13 in vivo. As shown in Fig. 6A, number of MMP13 positive cell was up-regulated whereas the number of collagen II positive cell was decreased in the DMM group compared to the sham group. Conversely, kirenol-treatment group markedly reversed this situation. In addition, the results of quantitative assessment showed the percentage of collagen II and MMP13 positive cells, in accordance with the macrographic changes (Fig. 6C).

**Fig. 7:**
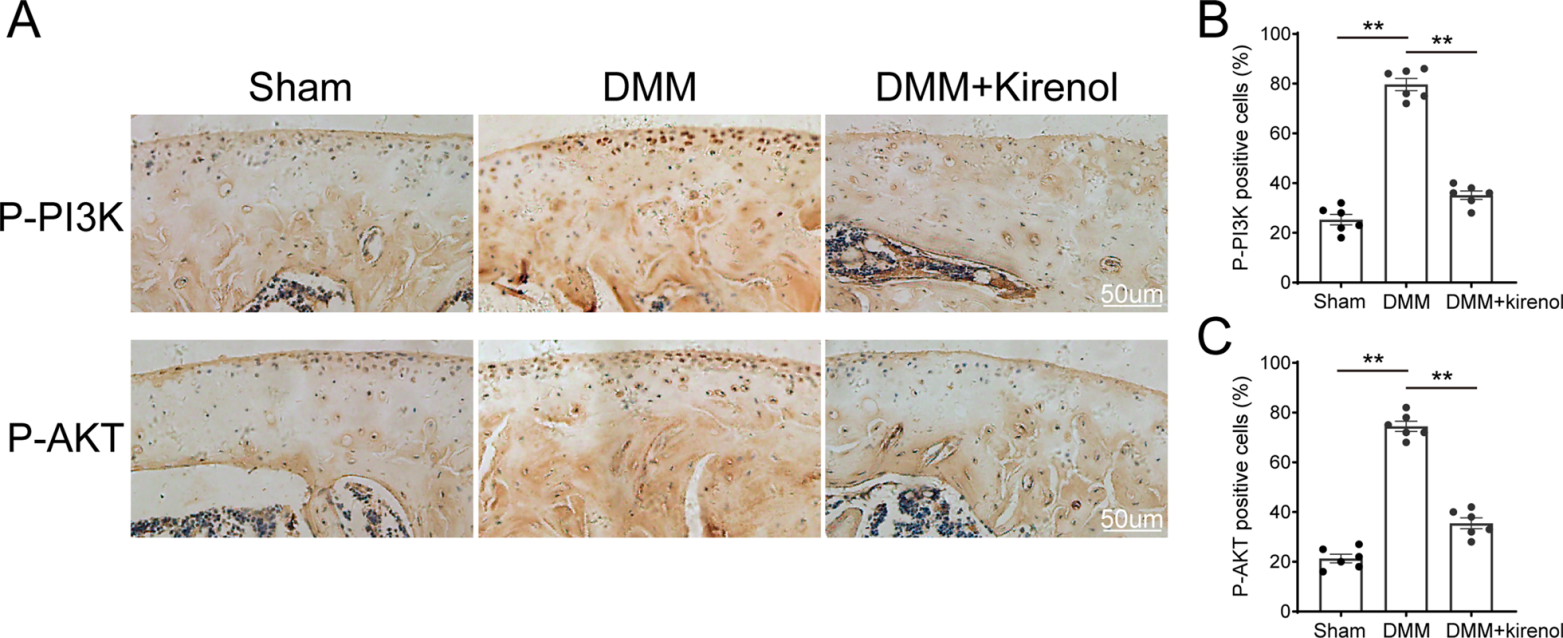
Effect of kirenol on PI3K/AKT pathway in vivo. **A** Immunohistochemical staining of p-PI3K, p-AKT expression in the cartilage samples (scale bar: 50 µm) (**B**, **C**). The percentages of p-PI3K and p-AKT positive cells in each section were quantified by Image Pro Plus. The data presented are the means ± S.D. *p < 0.05, and **p < 0.01, one-way AVOVA, *n* = 6 for immunohistochemical staining

To further demonstrate the potential molecular mechanism of kirenol in vivo, immunohistochemical staining was carried out to detect the expression of p-PI3K and p-AKT in the DMM mouse model. The result of immunohistochemical staining showed that sham group exhibited few areas of p-PI3K and p-AKT positivity. Conversely, the percentage of p-PI3K and p-AKT positive cells was increased in the DMM group, and this situation was markedly reversed in kirenol group (Fig. 7A). Furthermore, the results of quantitative analysis exhibited the percentage of p-PI3K and p-AKT positive cells, in line with the macrographic outcomes (Fig. 7B, C).

Taken together, these data above demonstrated that kirenol may inhibit the progression of OA in vivo.

## Discussion

Osteoarthritis is one of the most common aging-related joint disease, having a negative influence on the quality of life, even causing the major concern of public health with the continuously increasing incidence [24–26]. Among a variety of risk factors of OA, inflammation has been considered a key factor [6]. Currently, treatments in the clinic mainly focus on the symptoms relieving and progress controlling at the early stage, including the application of nonsteroidal anti-inflammatory drugs (NSAIDs) and hyaluronic acid injection into the articular cavity [27, 28]. At the late stage, there is no obviously effective treatment to delay the progression of disease other than operative treatment. However, commonly used drugs of NSAIDs relieved the clinical symptoms temporarily and may cause several side effects [29]. Thus, there is an urgent need to search for more effective agents with less side effects for better treatment options of OA.

Natural plants and traditional herbs play a significant role in treating many acute and chronic diseases. Kirenol, a diterpenoid natural product compound isolated from Herba Siegesbeckia, has been prove to exert various biological properties, including anti-inflammation, anti-apotosis, anti-oxidant property and anti-cancer effects, which has been demonstrated therapeutically effective in the inflammation related diseases, including wound healing, cardiovascular disease and numerous types of cancers [16–18, 20]. However, potential effect of kirenol and its mechanism have not been well explored in the progression of OA. Thus, this study aimed to understand the mechanisms involved in promoting the therapeutic effects of kirenol in OA. Not surprisingly, we illuminated that kirenol was able to restrain IL-1β-mediated inflammation response and ECM degradation in chondrocytes and its potential mechanism may attribute to NF-κB pathway regulation. Moreover, in vivo study also showed that kirenol could strongly mitigate the development of OA.

Previous studies have demonstrated that NF-κB pathway plays an important role in the regulation of inflammatory response, which is strongly involved in the pathogenesis and progress of OA [30–32]. IL-1β stimulation could induce the phosphorylation of IκBα, and then causes IκBα degradation in the cytoplasm and p65 phosphorylation, subsequently leading to the translocation of p65 to the nucleus from the cytoplasm. p65 in the nucleus could increase the synthesis of catabolic genes and pro-inflammatory mediators [33, 34]. NO is one of the important inflammatory mediators, which is catalyzed by iNOS, and increases the expression of MMPs and further inhibits the synthesis of collagen II and proteoglycan, ultimately inducing the ECM degradation [35]. PGE-2 is another inflammatory factor and its generation is induced by COX-2-mediated endogenous arachidonic acid, which could promote the activation of MMPs and ADAMTS5, and ECM catabolism [36]. These pro-inflammatory mediators above, combined with IL-6 and TNF-α, contribute strongly to the progression of OA. MMP13, one of the subgroup of collagenases, exerts its effect through irreversibly degradation of collagen II [37]. In addition, ADAMTS5 has been reported a primary aggrecanase targeting aggrecan’s cleavage, which is an important factor in the pathogenesis of OA [38].

**Fig. 8:**
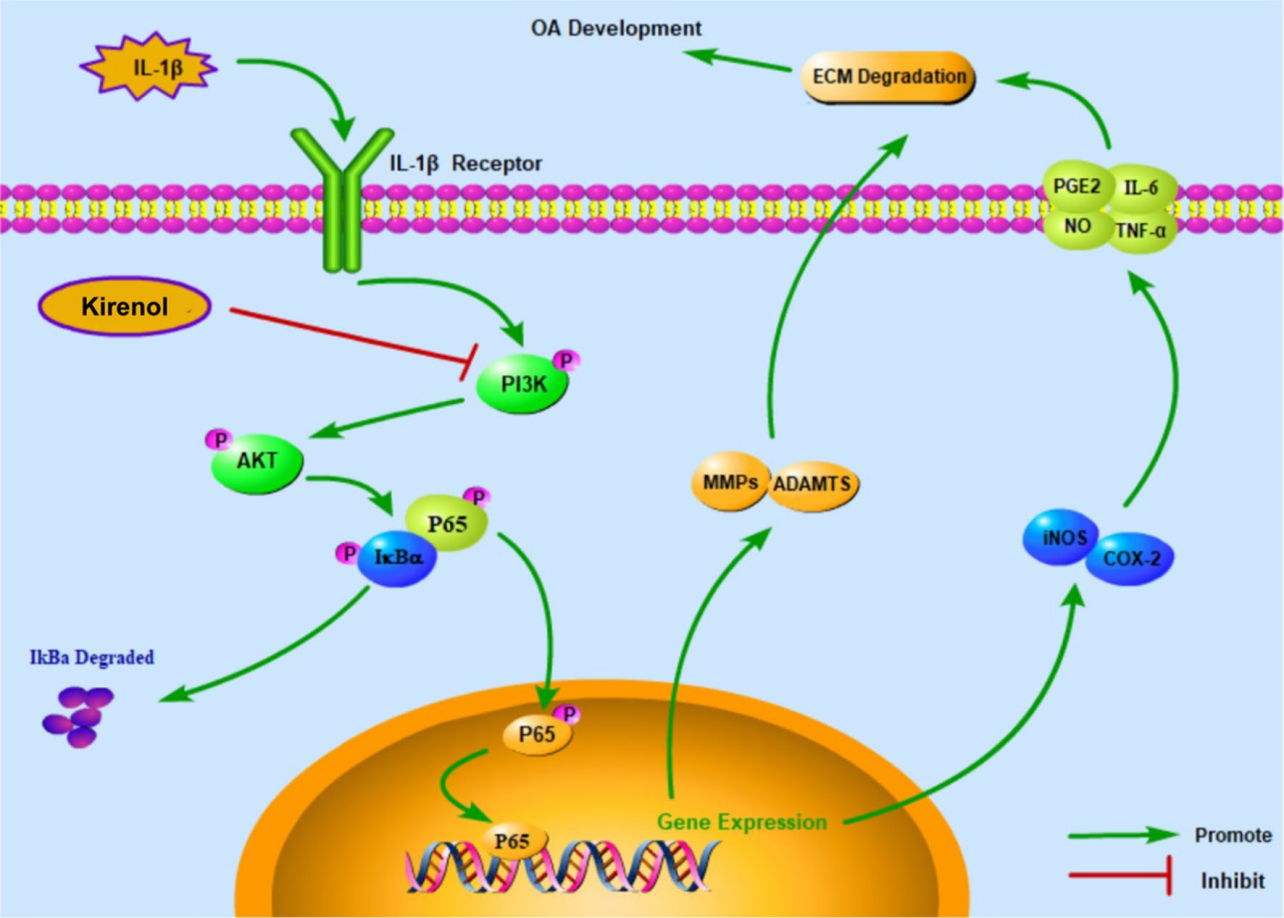
Schematic illustration of PI3K/AKT/NF-κB suppression by kirenol and its potential protective effects in osteoarthritis development. Red arrows indicate the inhibiting effect. Green arrows indicate the promoting effect

Besides, it is widely accepted that PI3K/Akt pathway is one of the crucial upstream signaling of the NF-κB signaling pathway and responsible for NF-κB pathway activation, which is implicated in pathogenesis and progression of OA [39]. In this study, the results showed that kirenol strongly inhibited the expression of PGE2, NO, IL-6, TNF-α as well as the COX-2 and iNOS upregulation after IL-1β stimulation. Moreover, we found that kirenol could mitigate the excessive generation of ADAMTS5 and MMP13, and the degradation of collagen II and aggrecan in IL-1β-stimulated chondrocytes. From these findings, kirenol could significantly alleviate the IL-1β-induced inflammation response and exert its effects through the regulation of PI3K/AKT/NF-κB pathway. Meanwhile, the potential molecular mechanism of kirenol was specifically present in the Fig. 8. However, this was only one part of the molecular mechanism of kirenol during OA development and exact regulatory mechanism are required to be further clarified in the future research. Although there may be additional upstream or bypass target, our work illuminated that kirenol could inhibit the activation NF-κB to some extent.

DMM was considered a reliable and effective animal model for in vivo experiments. During our study, DMM group showed serious cartilage destruction, calcification and apparent proteoglycan loss compared to the sham group. However, kirenol treatment could mitigate these harmful phenomena in DMM mice model, revealing the protective role of kirenol during OA progression. Furthermore, the activation of PI3K/AKT pathway was also inhibited following kirenol treatment in DMM mice model.

## Conclusions

In the current research, we provided potent evidence that kirenol may alleviate IL-1β-indued inflammatory response through restraining the activation of PI3K/AKT/NF-κB pathway. Furthermore, our study also indicated that kirenol was able to mitigate OA progression in surgical-induced DMM mouse model in vivo. In brief, our findings above illuminated that kirenol may serve as a promising therapeutic agent in prevention and treatment of OA.

## Data Availability

The data used to support the findings of this study are available from the corresponding author upon request.

## Abbreviations

OA: Osteoarthritis
IL-1β: Interleukin-1β
NO: Nitric oxide
PGE2: Prostaglandin E2
ADAMTS: Thrombospondin motifs
MMPs: Matrix metalloproteinases
ECM: Extracellular matrix
NF‐κB: Nuclear factor kappa B
DMSO: Dimethylsulfoxide
DAPI: 4', 6-Diamidino-2-phenylindole
FBS: Fetal bovine serum
WT: Wild-type
DMM: Destabilizing the medial meniscus
RIPA: Radioimmunoprecipitation assay
PMSF: Phenylmethylsulfonyl fluoride
ECL: Enhanced chemiluminescence
RT-PCR: Real-time polymerase chain reaction
EDTA: Ethylenediaminetetraacetic acid
SO: Safranin O-fast green
OARSI: Osteoarthritis Research Society International
SD: Standard deviation
ANOVA: One-way analysis of variance
